# The Association between Leptin Level and Breast Cancer: A Meta-Analysis

**DOI:** 10.1371/journal.pone.0067349

**Published:** 2013-06-27

**Authors:** Jingping Niu, Le Jiang, Weiheng Guo, Liang Shao, Yi Liu, Liqin Wang

**Affiliations:** 1 Department of Epidemiology and Statistics, School of Public Health, Hebei Medical University, Hebei, Shijiazhuang, China; 2 Department of Toxicology, School of Public Health, Hebei Medical University, Shijiazhuang, Hebei, China; 3 Department of Vehical Inspection, Zhuhai Entry-Exit Inspection and Quarantine Bureau, Zhuhai, Guangdong, China; Baylor College of Medicine, United States of America

## Abstract

**Background:**

Contradictory results have been reported regarding the association between leptin level and breast cancer. Therefore, a meta-analysis was performed to investigate this issue.

**Methods:**

Published literature from PubMed and the Chinese National Knowledge Infrastructure (CNKI) Database was retrieved. This study was performed based on different cases and control groups. The combined effect (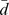) with 95% confidence interval (*CI*) was calculated using fixed-effects or random-effects model analysis.

**Results:**

Overall, the mean serum leptin level of case groups was significantly higher than that of control groups. A) For 9 studies comparing breast cancer cases and healthy controls the combined effect 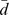 was 0.58 with 95% *CI* (0.48, 0.68). B) For 4 studies comparing premenopausal breast cancer cases and healthy controls the 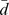 was 0.32 (0.12, 0.52). C) For 5 studies comparing postmenopausal cases and healthy controls the 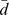 was 0.65 (0.46, 0.84). D) For 4 studies comparing breast cancer cases and breast benign controls the 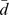 was 0.38 (0.17, 0.59). E) For 2 studies comparing premenopausal breast cancer cases and breast benign controls the 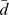 was 0.33 (-0.25, 0.91). F) For 6 studies comparing postmenopausal breast cancer cases and breast benign controls the 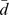 was 0.39 (0.19, 0.60). G) For 4 studies comparing lymph node metastasis positive cases and negative controls the 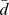 was 0.72 (0.45, 1.00). H) For 3 studies comparing breast benign cases and healthy controls the 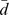 was 0.71 (0.41, 1.01).

**Conclusion:**

This meta-analysis suggests that leptin level plays a role in breast cancer and has potential for development as a diagnostic tool.

## Introduction

Breast cancer is the most common cancer and the second-leading cause of cancer related death among women worldwide [1]. Some risk factors have been identified and quantified, such as age, family history of breast cancer, marital status, early menarche, late menopause and the use of oral contraceptives [2]–[3]. The pathophysiology of breast cancer is highly complex, multifactorial, and far from being completely understood. Epidemiological studies have shown that obesity and weight gain might lead to increased risk of breast cancer in postmenopausal women [4]. However, the mechanism of how obesity relates to the development of breast cancer remains unclear.

Leptin, a protein hormone produced mainly by adipocytes, placenta and mammary epithelium, plays a significant role in the control of metabolism, reproductive processes, immune processes, angiogenesis, haemopoiesis and oxidation of lipids [5]. Leptin enhances breast cancer cell proliferation by inhibiting pro-apoptosis signalling pathways and by favouring in vitro sensitivity to oestrogens [6]. Leptin may also promote mammary tumor growth through multiple mechanisms such as modulation of the extracellular environment, down-regulation of apoptosis and/or up-regulation of anti-apoptotic genes [6]. The association between polymorphism of obesity-related genes (LEP, LEPR and PON1) and breast cancer risk has been investigated [7]–[9]. Liu and coworkers [7] suggested that the LEPR Q223R polymorphism might be implicated in the development of breast cancer in East Asians and PON1 L55M might increase breast cancer risk. He’s group [8] also suggested that LEPR Gln223Arg might be a low-penetrant risk for developing breast cancer, especially for black African women. LEPR polymorphisms rs1137101 and rs1137100 were found to be significantly correlated with breast cancer risk; while LEPR polymorphisms rs8179183, rs4655537 and rs3762274 displayed no association with breast cancer [9]. Therefore, it appears that leptin might influence the development of breast cancer.

Many studies have investigated the association between serum leptin level and breast cancer. However, the reported results have been contradictory. In this study, we performed a meta-analysis to assess the association between serum leptin level and breast cancer risk.

## Methods

### Search Strategy

To identify all studies that examined the relationship between leptin level and breast cancer, a systematic online databases search was conducted. The search was done on June 10, 2012. All published studies were found with PubMed/MEDLINE and China National Knowledge infrastructure (CNKI) using the following terms: (“leptin” or “ob gene product” or “ob protein” or “obese gene product” or “obese protein” ) and (“breast cancer” or “breast carcinoma” or “breast tumor” or “breast neoplasm” or “breast tumors” or “breast neoplasms” or “mammary carcinoma” or mammary neoplasm” or “mammary neoplasms” or “cancer of breast” or “cancer of the breast” or “human mammary carcinoma”). References from the retrieved articles were also screened to complete the data bank. No “language”, “publication year”, or other limits were used. If more than one article were published using the same case series, only the study with largest sample size was selected.

### Selection Criteria and Data Extraction

Studies were eligible for inclusion if: (1) They could be defined as a case-control study or nested case-control study or a cohort study, (2) all patients were pathological diagnosed with breast cancer that had not undergone any previous treatment for tumors, (3) they included sufficient data for determining serum leptin level. We excluded studies that were not published as full reports, or studies without control subjects, or studies that included patients who received antitumor treatments.

With the purpose of extracting the necessary characteristics, all relevant articles were collated independently by two reviewers (Jingping Niu and Liqin Wang). They checked for any encountered discrepancies and reached a consensus.

The extracted data included: (1) publication details: first author’s last name, publication year, and origin of the studied population, (2) study design, (3) source of control subjects, (4) characteristics of the studied population: sample size, mean of age, mean of BMI, (5) methods of leptin measurement, and (6) means and standard deviation (*SD*) of leptin level in each group. If standard error of mean (*SEM*) was reported, *SD* was calculated according to the formula: 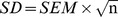.

### Statistical Analysis

All analyses were performed using Review Manager (version 5.1.2) and STATA (version 11.0). For comparing data from different sources, the absolute difference of leptin levels (mean difference (*MD*)) was converted into standard mean difference (*SMD*). The combined effect (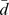) with 95% *CI* was calculated in a fixed- or random-effect model (as shown in Formulas S1) to assess the strength of the association between serum leptin level and breast cancer.

We used the *I* ^2^ statistic to investigate heterogeneity among studies [10]–[11]. If there was a statistical difference in terms of heterogeneity (*P*≤0.10), a random-effect model was selected to pool the data. Otherwise, a fixed-effect model was used.

To investigate the sources of heterogeneity, meta-regression analyses were conducted [12]–[13]. If meta-regression could not explain the sources of heterogeneity the studies generating heterogeneity according to Galbraith bar were removed to obtain more reliable results. If meta-regression could not be performed due to the small number of eligible studies, these studies were excluded according to Galbraith bar. Publication bias was evaluated using the funnel plot and the Egger test [14]. Fail-safe numbers were calculated to estimate stability of the results [15].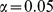.

## Results

### Study Characteristics

**Figure 1:**
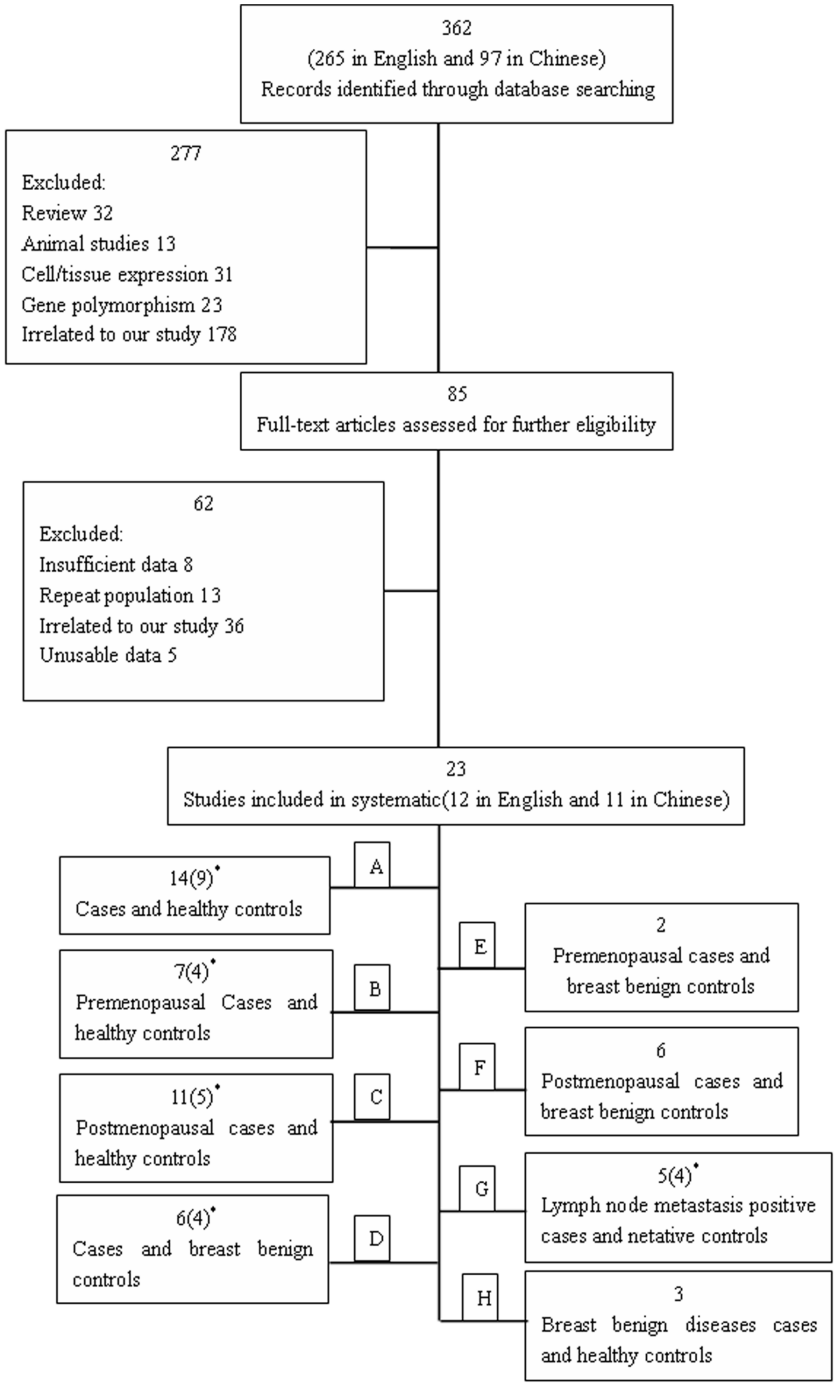
Flow chart of meta-analysis for exclusion/inclusion of studies. ^*^Number of studies with no heterogeneity.

The flowchart summarizing the process of study search and selection is presented in Figure 1. A total of 362 relevant studies were retrieved from the initial literature search. Following the subsequent selection, 23 studies were included in our meta-analysis. Key characteristics of these studies are reported in Table 1. 2058 breast cancer patients, 2078 healthy controls and 285 breast benign controls were screened for the 23 studies included in this meta-analysis. These studies were classified into 8 groups according to different cases and controls: (A) breast cancer cases and healthy controls (14 studies involving 1338 patients and 1335 controls) [16]–[29]; (B) premenopausal breast cancer cases and healthy controls (7 studies involving 323 patients and 291 controls) [16]–[18], [26], [30]–[32]; (C) postmenopausal cases and healthy controls (11 studies involving 698 patients and 672 controls) [16]–[18], [26], [30]–[36]; (D) breast cancer cases and breast benign controls (6 studies involving 529 patients and 234 controls) [19]–[20], [22], [30], [37]–[38]; (E) premenopausal breast cancer cases and breast benign controls (2 studies involving 92 patients and 52 controls) [30], [38]; (F) postmenopausal breast cancer cases and breast benign controls (6 studies involving 355 patients and 154 controls) [30], [33]–[35], [37]–[38]; (G) lymph node metastasis positive cases and negative controls (5 studies involving 179 patients and 141 controls) [19]–[21], [33]–[34]; and (H) breast benign diseases cases and healthy controls (3 studies involving 85 patients and 101 controls) [19]–[20], [22]. (Fig. 1). The results of the meta-analysis are summarized in Table 2.

**Table 1:** Characteristics of the studies included in the meta-analysis.

				Cases					Controls		
Author	Year	Country	Leptin Measurement method	*N*	Age	BMI		*N*		Age	BMI
Mantzoros	2003	Greece	RIA	174	63.1	26.0	167			62.2	25.6
Tessitore	2003	sweden	RIA	49	57.7	24.6	12			57.2	23.8
Jen	2005	USA	RIA	165	57.4	29.5	155			55.0	28.2
Ju-Xing Gao	2005	China	RIA	74	NG	24.8	30			NG	21.6
Xiu-Ming Wang	2005	China	RIA	64	NG	23.6	31			NG	20.3
Hua Yu	2005	China	RIA	46	59.8	23.6	41			61.3	21.8
Chen	2006	China	RIA	100	49.9	22.9	100			48.9	23.8
Woo	2006	korea	RIA	30	NG	NG	26			NG	NG
Li-Li Du	2006	China	RIA	90	49.5	25.1	103			46.6	23.4
Xu-Dong Huang	2006	China	RIA	36	53.1	NG	56			58.3	NG
Dong-Lin Jiang	2006	China	ELISA	68	58.4	25.1	40			56.5	23.2
Jun-Ming Sun	2006	China	RIA	55	56.7	24.8	20			51.1	22.7
Liu	2007	China	ELISA	47	50.9	23.8	41			47.6	21.9
Pazaitou	2007	Greece	ELISA	74	62.5	29.1	76			55.6	29.3
Han	2008	China	ELISA	240	45.0	25.1	500			44.0	23.4
Aliustaoglu	2009	Turkey	ELISA	30	53.0	27.2	30			40.4	27.3
Hong-Xia Cui	2009	China	ELISA	68	55.0	26.2	62			52.0	23.6
Xiu-Li Fan	2009	China	ELISA	98	46.0	24.8	47			42.0	23.4
Hancke	2010	Germany	ELESA	40	NG	NG	25			NG	NG
Maccio	2010	Finland	ELESA	82	60.5	25.6	105			58.7	23.4
Yang Liu	2010	China	RIA	79	46.2	NG	60			45.1	NG
Dalamaga	2011	Greece	ELISA	102	61.5	27.7	102			62.8	25.9
Yan Wang	2011	China	ELISA	132	46.0	24.1	60			43.0	23.3

N: sample size; Age: mean age; BMI: mean body mass index; NG: Not given.

**Table 2:** Meta-analysis for all groups of leptin level.

Cases vs. controls	Reference	Model	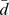 (95%*CI*)	*I* ^2^	*P*
BC vs. HC					
All studies(n = 14)	15–28	Random-effect	0.74(0.45, 1.03)	92	<0.001
Studies with no heterogeneity(n = 9)	18–25,28	Fixed-effect	0.58(0.48, 0.68)	33	0.15
Pre-BC vs. Pre-HC					
All studies(n = 7)	15–17,25,29–31	Random-effect	0.41(0.09,0.74)	72	<0.001
Studies with no heterogeneity(n = 4)	17,25,30–31	Fixed-effect	0.32(0.12, 0.52)	0	0.51
Post-BC vs. Post-HC					
All studies(n = 11)	15–17,25,29–35	Random-effect	1.09(0.57,1.62)	95	<0.001
Studies with no heterogeneity(n = 5)	25,29–31,34	Fixed-effect	0.65(0.46,0.84)	0	0.49
BC vs. BBC					
All studies(n = 6)	18–19,21,29,36–37	Random-effect	0.96(0.04,1.88)	97	<0.001
Studies with no heterogeneity(n = 4)	18–19,21,37	Fixed-effect	0.38(0.17,0.59)	0	0.88
Pre-BC vs. Pre-BBC					
All studies(n = 2)	29,37	Random-effect	0.33(-0.25,0.91)	65	0.09
Post-BC vs. Post-BBC					
All studies(n = 6)	29,32–34,36–37	Fixed-effect	0.39(0.19,0.60)	36	0.17
LN+ vs. LN-					
All studies(n = 5)	18–20,32–33	Random-effect	0.59(0.21,0.97)	62	0.03
Studies with no heterogeneity(n = 4)	18–19,32–33	Fixed-effect	0.72(0.45,1.00)	14	0.32
BBC vs. HC					
All studies(n = 3)	18–19,21	Fixed-effect	0.71(0.41,1.01)	0	0.95

BC;breast cancer; HC: healthy control; Pre-BC: premenopausal breast cancer; Pre-HC: premenopausal healthy control; Post-BC: postmenopausal breast cancer; Post-HC: postmenopausal healthy control; BBC;benign breast controls; Pre-BBC;premenopausal benign breast controls;Post-BBC: postmenopausal benign breast controls; LN+: lymph node metastasis positive cases; LN-: lymph node metastasis negative controls.

### Quantitative Data Synthesis

**Figure 2:**
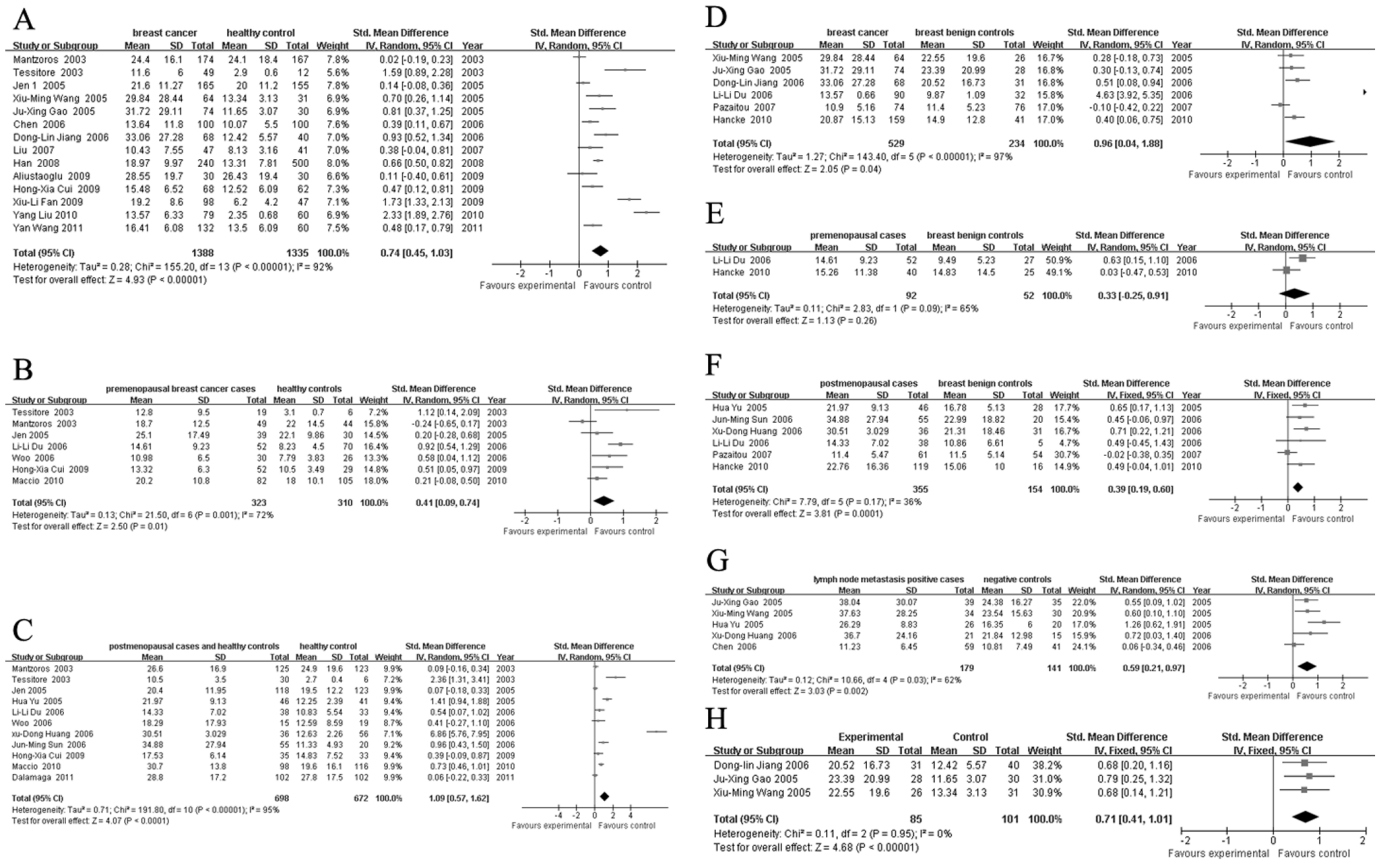
Forest plots for all groups with all included studies. A: breast cancer cases and healthy controls; B: premenopausal breast cancer cases and healthy controls; C: postmenopausal cases and healthy controls; D: breast cancer cases and breast benign controls; E: premenopausal breast cancer cases and breast benign controls; F: postmenopausal breast cancer cases and breast benign controls; G: lymph node metastasis positive cases and negative controls; H: breast benign diseases cases and healthy controls.

**Figure 3:**
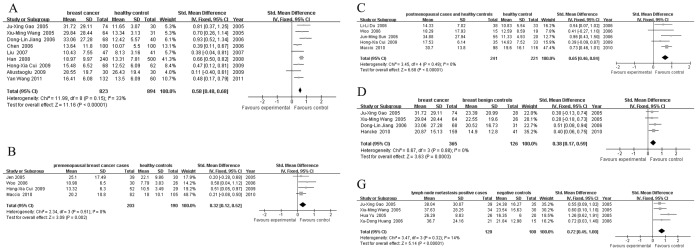
Forest plots for A B C D G groups of studies with no heterogeneity. A: breast cancer cases and healthy controls; B: premenopausal breast cancer cases and healthy controls; C: postmenopausal cases and healthy controls; D: breast cancer cases and breast benign controls; G: lymph node metastasis positive cases and negative controls.

**Figure 4:**
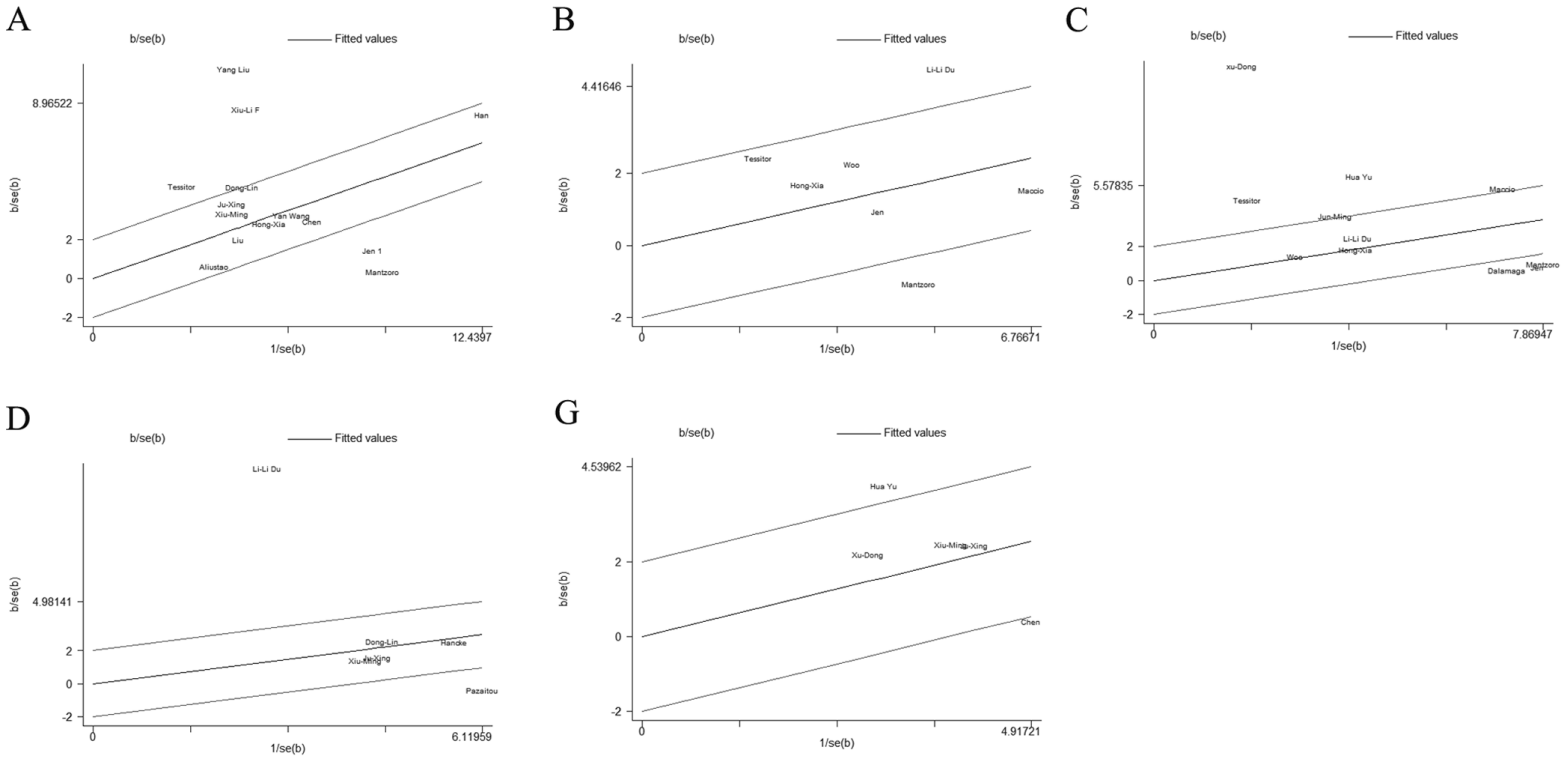
Galbraith bars for A B C D G groups with all included studies. A: breast cancer cases and healthy controls; B: premenopausal breast cancer cases and healthy controls; C: postmenopausal cases and healthy controls; D: breast cancer cases and breast benign controls; G: lymph node metastasis positive cases and negative controls.

Heterogeneities were found within studies of groups A, B, C, D, E and G (*I* ^2^ values were 92%, 72%, 95%, 97%, 65% and 62%, respectively; *P* values were <0.001, 0.001, <0.001, <0.001, 0.09 and 0.03, respectively). A random-effects model was applied for these groups. The combined effects 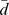 with 95% *CI* were A) 0.66 (0.39, 0.92), B) 0.41 (0.09, 0.74), C) 1.09 (0.57, 1.62), D) 0.96 (0.04, 1.88), E) 0.33 (-0.25, 0.91) and G) 0.59 (0.21, 0.97). Meta-regression did not show a significant difference in published year, sample size, age, or BMI. For group A, heterogeneity could be attributed mainly to five studies [16]–[18], [27]–[28] according to Galbraith bar. For group B, 2 studies [16], [30] contributed mainly to the heterogeneity. For group C, 6 studies [16]–[18], [33]–[34], [36] accounted for the heterogeneity. For groups D and G, 2 studies [30], [37] and 1 study [21] contributed to the heterogeneity respectively. After excluding these studies, homogeneity test showed no statistical significant differences (*I* ^2^ values were 33%, 0%, 0%, 0% and 14%, respectively; *P* values were 0.15, 0.51, 0.49, 0.88 and 0.32, respectively) in the remaining studies. The combined effect 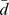 were 0.58 (0.48, 0.68), 0.32 (0.12, 0.52), 0.65 (0.46, 0.84), 0.38 (0.17, 0.59) and 0.72 (0.45, 1.00), respectively. Because of the limited literature quantity, heterogeneity sources of group E could not be determined by Galbraith bar. Overall, the leptin level was higher in patients. The results are presented in Fig. 2, Fig. 3 and Fig. 4.

There was no heterogeneity within studies of groups F and H (*I* ^2^ values were 36% and 0%, *P* values were 0.17 and 0.95). The combined effect 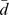 were 0.39 (0.19, 0.60) and 0.71 (0.41, 1.01). Overall, the leptin level was higher in patients. The results are presented in Fig. 2.

Fail-safe numbers of each group, indicating the publication bias, are reported in Table 3. When the meta-analysis results are statistically significant, the minimum number of unpublished studies (fail-safe number) can be calculated to reverse the conclusion or to bring the meta-analytic mean effect size down to a statistically insignificant level. The greater the fail-safe number is, the more stable the result is. The fail-safe numbers were all relatively large in our meta-analysis except for group E, suggesting that the results were reliable.

**Table 3:** Fail-safe numbers of all groups for studies with no heterogeneity.

		Fail-safe number	
Group	Number of study	α = 0.05	α = 0.01
A	9	344.098	165.933
B	4	9.635	2.755
C	5	70.489	32.399
D	4	15.591	5.706
E	2	0.908	−0.559
F	6	32.956	13.300
G	4	43.452	19.509
H	3	23.745	10.250

A: breast cancer cases and healthy controls; B: premenopausal breast cancer cases and healthy controls; C: postmenopausal cases and healthy controls; D: breast cancer cases and breast benign copone.0067349.g001.tifntrols; E: premenopausal breast cancer cases and breast benign controls; F: postmenopausal breast cancer cases and breast benign controls; G: lymph node metastasis positive cases and negative controls; H: breast benign diseases cases and healthy controls.

### Publication Bias

According to the funnel plot and Egger's test, publication bias was not detected for groups A, B, C, D, F, G and H (*P* values were 0.537, 0.218, 0.539, 0.655, 0.278, 0.303, 0.657, respectively). However, it was unable to estimate publication bias for group E due to the limited quantity of the primary studies.

## Discussion

Overall the results of this study suggest that the circulating leptin level varies among these different population groups from low to high: healthy people<breast benign diseases patients<breast cancer patients <lymph node metastasis positive patients.

The strength of the present study is that it is the first systematic review and meta-analysis, to our knowledge, to evaluate the relationship between serum leptin level and breast cancer. It is not recommended to perform meta-analysis when high heterogeneity exists [10]. We adequately evaluated the role of heterogeneity in our results. Heterogeneity was present particularly in groups A, B, C, D, and G. Our results could be divided into two parts. One part includes the results that met the criterion, the other part includes the results with no heterogeneity and publication bias. For group E, considering the small number of studies, it was not possible to estimate publication bias or homogeneity. There was no disagreement after removal of the studies contributing to heterogeneity. In summary, the results excluding studies with heterogeneity were reliable.

Several techniques were used to obtain more reliable results. First, only observational studies were included in this meta-analysis. Similar to other meta-analyses, the validity of the results depends on the originally screened studies. Thus, we applied strict criteria for the selection of studies to minimize potential errors. While a number of studies reported leptin level in breast cancer and controls using a frequency table [39], others reported results as medians and interquartile ranges instead of means and SDs [40]. We used studies that clearly provided means and SDs. Second, publication bias was minimized through the examination of a symmetric funnel plot and Egger’s test. These tests did not reveal any evidence of publication bias in any of the groups except for group B. Since publication bias may lead to unreliable results [41], we excluded the study generating publication bias.

Several deficiencies should also be considered. First, there was some language bias. We only screened studies in English and Chinese, so language bias may occur in our meta-analysis. Second, in our meta-analysis when comparing patients and controls, we failed to find potential explanations for heterogeneity, although we formulated strict inclusion and exclusion criteria. Third, there were only 2 studies involved in group E. Thus, heterogeneity and publication bias could not be avoided due to the limited literature quantity. The fail-safe number also suggested that the result was unstable. The result for group E should be interpreted with caution.

Results of this meta-analysis are consistent with a variety of observational and experimental studies that support a role for leptin in tumor progression. Ishikawa [42] showed that leptin may play a role in the carcinogenesis and metastasis of breast cancer, possibly in an autocrine manner. Garofalo [43] reported that high leptin level in obese breast cancer patients might contribute to the development of antiestrogen resistance. Moreover, results from animal studies suggested that leptin receptor antagonists might be a new option for breast cancer treatment [44].

Laboratory data at the molecular level support the present results. Furthermore, there have been some meta-analyses and systematic reviews that investigated the association between leptin receptor gene polymorphism and breast cancer [7]–[9]. These results were conflicting as we mentioned in the introduction. Grossmann [45] indicated that a high adiponectin to leptin ratio is indicative of a positive risk profile compared to a low adiponectin to leptin ratio. Rose [46] suggested that leptin might be a strong candidate for a role as a proximate effector in mediating the adverse influence of obesity on breast cancer prognosis. In this study, we focused on the relationship between leptin level and breast cancer. The results indicated that higher leptin may be associated with increased incidence and development of breast cancer.

For group A, among the 9 studies without heterogeneity, 7 studies reported positive results with statistical significance. The other 2 studies displayed higher leptin level in breast cancer patients than that of healthy controls, although no statistical significance was shown. The results for group B and C were similar to group A. The leptin level of breast cancer patients for group B and C was higher than that of healthy controls, regardless of menopausal status. Some differences were statistically significant, while some were not. The results from groups A, B and C indicated that elevated leptin levels may promote breast cancer. Therefore, leptin level is proposed as a screening tool in groups with high breast cancer risk. For groups D and F, the leptin level was higher in breast cancer patients than that of breast benign controls. However, no statistical significance was found in group E. Limited literature quantity and existence of heterogeneity might account for this. Though not significant, the leptin level was found to be higher in breast cancer patients of group E. Therefore, it might be possible to distinguish breast cancer and breast benign diseases by evaluating leptin level. For group G, lymph node metastasis positive cases displayed higher leptin level; and all the individual study reported positive results. Thus, higher leptin level may indicate a poor prognosis. For group H, the leptin level was higher in breast benign diseases patients than in healthy controls. Overall, the leptin level of the four population can be ranked as follows: healthy controls<breast benign diseases patients<breast cancer patients<lymph node metastasis positive breast cancer patients. However, the boundary values require further effort to establish.

In summary, leptin might play a role in the formation and development of breast carcinoma as well as prognosis. However, the underlying mechanisms remain unclear and require further in-depth study on macro and micro levels. In addition, the reference range of leptin level in a normal population should be determined. More data from longitudinal studies is required to clarify the relationships between serum leptin level and breast cancer risk. We believe that, with further investigation on leptin, new medications could be developed to combat breast cancer and other breast diseases.

## Supporting Information

Checklist S1 **PRISMA Checklist.**
(DOC)

Text S1 **PRISMA Flow Diagram.**
(DOC)

Text S2
**Calculation formalas for SMD and combined effect (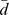) with 95% CI.**
(DOC)
