# Supplementary material for: The Association between Leptin Level and Breast Cancer: A Meta-Analysis
Source: PLoS One. 2013 Jun 27;8(6):e67349. doi: 10.1371/journal.pone.0067349 (PMC3694967; doi:10.1371/journal.pone.0067349)
Supplement: Text S1 — PRISMA Flow Diagram. (DOC) [file pone.0067349.s002.doc]

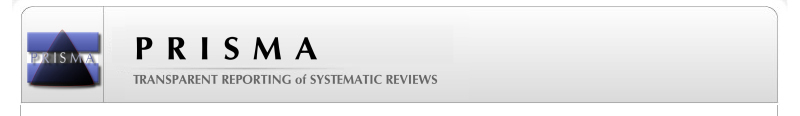
**PRISMA 2009 Flow Diagram**

**Screening**

**Included**

**Eligibility**

**Identification**

Records identified through database searching
(*n* = 362)

Additional records identified through other sources
(*n* = 0)

Records after duplicates removed
(*n* =13)

Records screened
(*n* = 349)

Records excluded
(*n* = 277)

Full-text articles assessed for eligibility
(*n* = 72)

Full-text articles excluded, with reasons
(*n* = 49)

Studies included in qualitative synthesis
(*n* = 23)

Studies included in quantitative synthesis (meta-analysis)
(*n* = 23)
