# Supplementary material for: The Association between Leptin Level and Breast Cancer: A Meta-Analysis
Source: PLoS One. 2013 Jun 27;8(6):e67349. doi: 10.1371/journal.pone.0067349 (PMC3694967; doi:10.1371/journal.pone.0067349)
Supplement: Text S2 — Calculation formalas for SMD and combined effect () with 95% CI. (DOC) [file pone.0067349.s003.doc]

**1. Combined effect of fixed-effect model**

1. Single study effect

Standardized mean difference (*SMD*)：Because the data retrieved from the literature have different units of measurement, *SMD* was employed in our meta-analysis to compare the effect between control and experimental groups.

$$d_{i}=\frac{\overline{X}_{1i}-\overline{X}_{2i}}{S_{i}}$$

1. The formulas for calculating the combined effect ($\delta$) of weight (*w*_i_), weighted mean ($\bar{d}$) and its standard deviation ($S_{\bar{d}}$) are as follows:

$$w_{i}=n_{1i}+n_{2i}$$

$$\bar{d}=\frac{\sum w_{i}d_{i}}{\sum w_{i}}$$

$$S_{\bar{d}}=\sqrt{\frac{1}{\sum w_{i}}}$$

1. The 95% confidence interval (95% *CI*) for the combined effect $\delta$ would be computed as: $\bar{d}\pm1.96S_{\bar{d}}$

If “0” is involved in 95% *CI*, it suggests that no statistical significance is found between control and experimental groups.

If “0” is not involved in 95% *CI*, it suggests that statistical difference is existed between control and experimental groups.

1. Z-score for combined effect:$Z=\frac{\bar{d}}{S_{\bar{d}}}$

If $P\leq\alpha$（$\alpha$=0.05），then reject *H*_0_, accept *H*_1_, it should be taken into consideration that the mean of the combined effect size might not equal to 0; if $P>\alpha$，then *H*_0_ is not rejected，it should not be taken into consideration that the mean of the effect size might not equal to 0.

**2. Combined effect of random-effect model**

1. Single study effect: same as fixed-effect model
2. The formulas for calculating combined effect ($\delta_{DL}$) of weight ($w_{i}^{'}$), weighted mean ($\bar{d}_{DL}$) and its standard deviation（$S_{\bar{d}_{DL}}$）are as follows:

$$w_{i}^{'}=\frac{1}{\frac{1}{w_{i}}+\tau^{2}}$$

$$w_{i}=n_{1i}+n_{2i}$$

$$\tau^{2}=\left\{\begin{matrix} \frac{Q-(k-1)\sum w_{i}}{(\sum w_{i})^{2}-\sum w_{i}^{2}}\begin{matrix} & & & \text{ }Q\geq k-1 \end{matrix} \\ \begin{matrix} \begin{matrix} 0 & & & \end{matrix} & & & \begin{matrix} \begin{matrix} & \end{matrix} & \end{matrix}Q<k-1 \end{matrix} \end{matrix}\right.$$

$\tau^{2}$is the within-study variance (random effect).

$$\bar{d}_{DL}=\frac{\sum w_{i}^{'}d_{i}}{\sum w_{i}^{'}}$$

$$S_{\bar{d}_{DL}}=\frac{1}{\sum w_{i}^{'}}$$

1. The 95% confidence interval (95% *CI*) for the combined effect （$\delta_{DL}$） would be computed as:：$\bar{d}_{DL}\pm1.96S_{\bar{d}_{DL}}$
2. Z-score for combined effect:：$Z=\frac{\bar{d}_{DL}}{S_{\bar{d}_{DL}}}$

If $P\leq\alpha$（$\alpha$=0.05），then reject *H*_0_, accept *H*_1_, it should be taken into consideration that the mean of the combined effect size might not equal to 0; if $P>\alpha$，then *H*_0_ is not rejected，it should not be taken into consideration that the mean of the effect size might not equal to 0.
